# Current and novel therapeutic strategies for optimizing immunotherapy outcomes in advanced non-small cell lung cancer

**DOI:** 10.3389/fonc.2022.962947

**Published:** 2022-12-08

**Authors:** Claudio Martin, Diego Enrico

**Affiliations:** ^1^ Department of Medical Oncology, Thoracic Oncology Section, Alexander Fleming Cancer Institute, Buenos Aires, Argentina; ^2^ Department of Clinical Research, Alexander Fleming Cancer Institute, Buenos Aires, Argentina

**Keywords:** non-small-cell lung cancer, immunotherapy, immune checkpoint inhibitors, combinations, chemotherapy, antiangiogenic, co-inhibitory

## Abstract

During the past decade, immunotherapy has dramatically improved the outcomes of patients with non-small cell lung cancer (NSCLC). The development of specific antibodies against the programmed death (PD1) receptor and its ligand PD-L1 (programmed death ligand-1) has demonstrated substantial efficacy in advanced NSCLC either in the first or in the second line. However, the success of immune checkpoint inhibitors (ICIs) as monotherapy did not reach all patients and long-term responders still represent a small subset of cases. Under these circumstances, different strategies have been and are being tested to optimize clinical outcomes. Here, we reviewed the current evidence and the more promising perspectives of ICI combination approaches, such as the addition of chemotherapy, antiangiogenic agents, other co-inhibitory or co-stimulatory checkpoints, and targeted therapies.

## Introduction

During the past decade, the advent of immunotherapy has dramatically changed the outcomes of patients with non-small cell lung cancer (NSCLC) (1). The growing understanding of the environment in which tumor and immune cells interact led to the discovery of immune checkpoint inhibitors (ICIs) that block inhibitory pathways that physiologically control the immune response driving to restore and sustain the immune system against cancer cells (2).

Under this circumstance, the development of specific antibodies against the programmed death (PD1) receptor and its ligand PD-L1 (programmed death ligand-1) has led to a change of paradigm in the therapeutic strategies of advanced NSCLC either in the first- or in the second-line setting. Importantly, these drugs have unprecedented prolonged survival for a substantial proportion of these patients (3). However, not all NSCLCs respond appropriately to ICI as monotherapy, and long-term responders still represent a limited group that is challenging to find and predict. The objective response rate when using first-line single-agent ICI treatment is below 45% in highly biomarker-selected NSCLC patients such as PD-L1 expression (4). Furthermore, 40% to 60% of patients experienced disease progression within the first 6 months of treatment. Of note, this situation differs substantially from those reported for the efficacy of targeted therapy in oncogene-addicted NSCLC (5).

In this context, we are now in a race to find different strategies to optimize the efficacy of immunotherapy in lung cancer. The recent understanding of *de novo* or adaptive resistance, as well as the mechanisms involved in the induction of an effective antitumor immune response, provides the rationale for several established and novel ICI combination approaches such as the addition of chemotherapy, antiangiogenic agents, other immunotherapy, or targeted therapies. Here, we reviewed the current evidence and the more promising perspectives in this field.

## First-line combinations with chemotherapy

**Figure 1:**
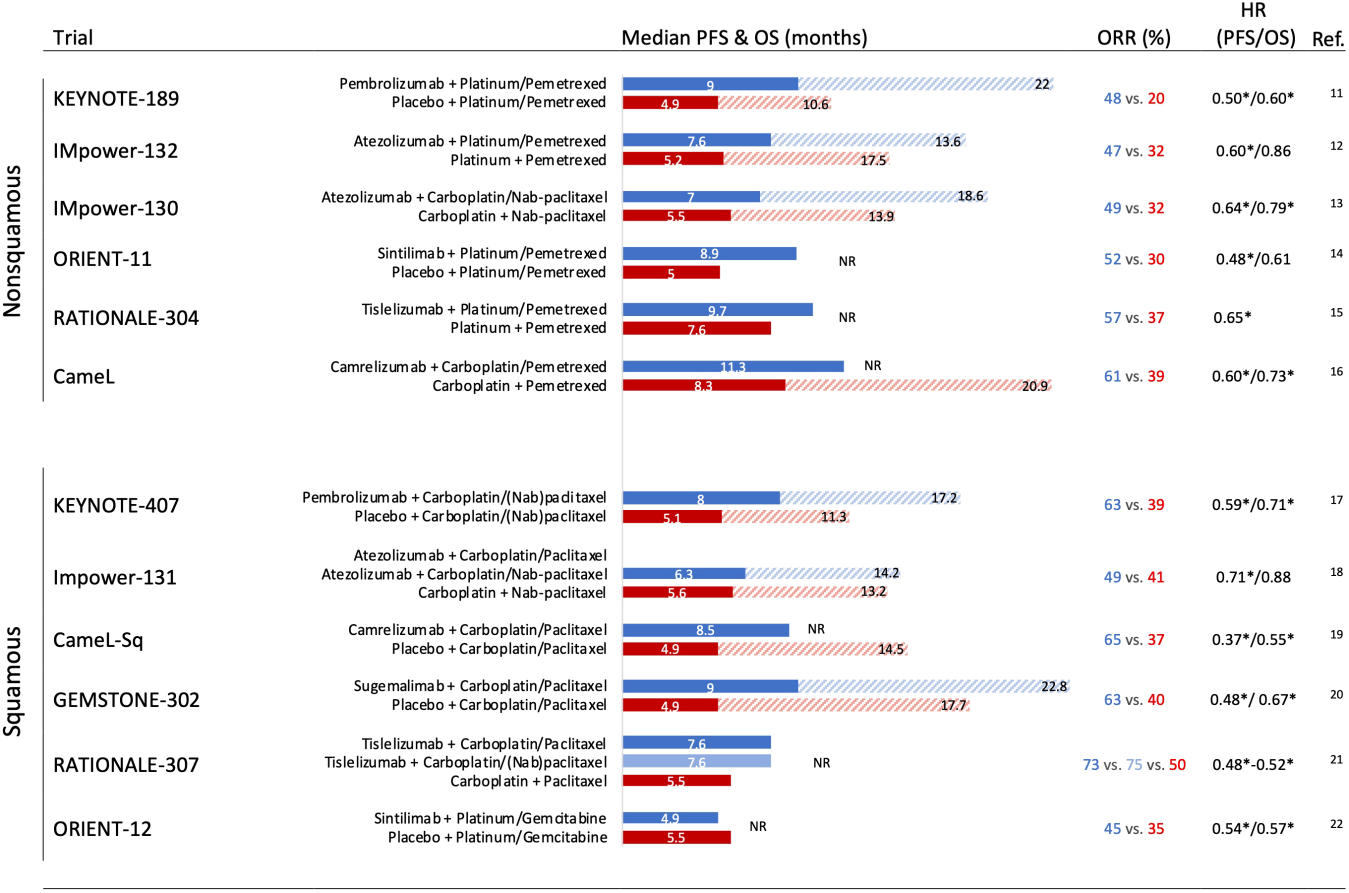
Phase III trials assessing an immune checkpoint inhibitor + chemotherapy strategies in the first-line setting in nonsquamous and squamous non-small cell lung cancer with outcomes. HR, hazard ratio; OS, overall survival; PFS, progression-free survival; ORR, overall response rate; NR, not reached (overall survival). ^*^ Significant improvement.

It has been demonstrated that modulation of the immune response through PD-1 inhibition may be enhanced by the synergistic immunogenic effects of cytotoxic chemotherapy by different mechanisms, including increasing the potential for antigen cross-presentation by dendritic cells after the destruction of tumor cells, induction of proinflammatory cytokines, inhibition of myeloid-derived suppressor cells, and induction of PD-L1 expression on tumor cells (6–10). Following this rationale, the combination of chemotherapy plus ICI has been tested in several NSCLC phase III clinical trials in the first-line setting. Notably, this approach has shown substantial efficacy when compared with platinum-based chemotherapy in unselected PD-L1 expression for both histology tumors among phase III clinical trials in the first-line scenario (**Figure 1**) (11–22). The addition of chemotherapy to ICI reported global overall response rates (ORRs) between 45% and 75%. Across all the trials, the immune-chemotherapy strategy significantly prolonged the median progression-free survival (PFS) compared with chemotherapy, showing safety and a generally manageable toxicity profile. However, overall survival (OS) improvement was not consistent in all the studies. Impower-131 and Impower-132 trials did not demonstrate a statistically significant difference in the intention-to-treat OS analysis, potentially explained by subsequent second-line treatments, percentage of PD-L1 tumor expression, patient population selection, overperformance of comparators arms, and possible differences across PD-1 and PD-L1 treatments.

## First-line immunotherapy combinations

PD-1 and cytotoxic T-lymphocyte-associated protein 4 (CTLA-4) are complementary coinhibitory receptors that modulate T-cell responses (23). Thus, using antibodies to blockade both receptors simultaneously has been fruitful in many tumor types, including melanoma, renal cell carcinoma, malignant pleural mesothelioma, esophageal squamous cell carcinoma, microsatellite instability-high colorectal cancer, hepatocellular carcinoma, and NSCLC (24–30).

**Figure 2:**
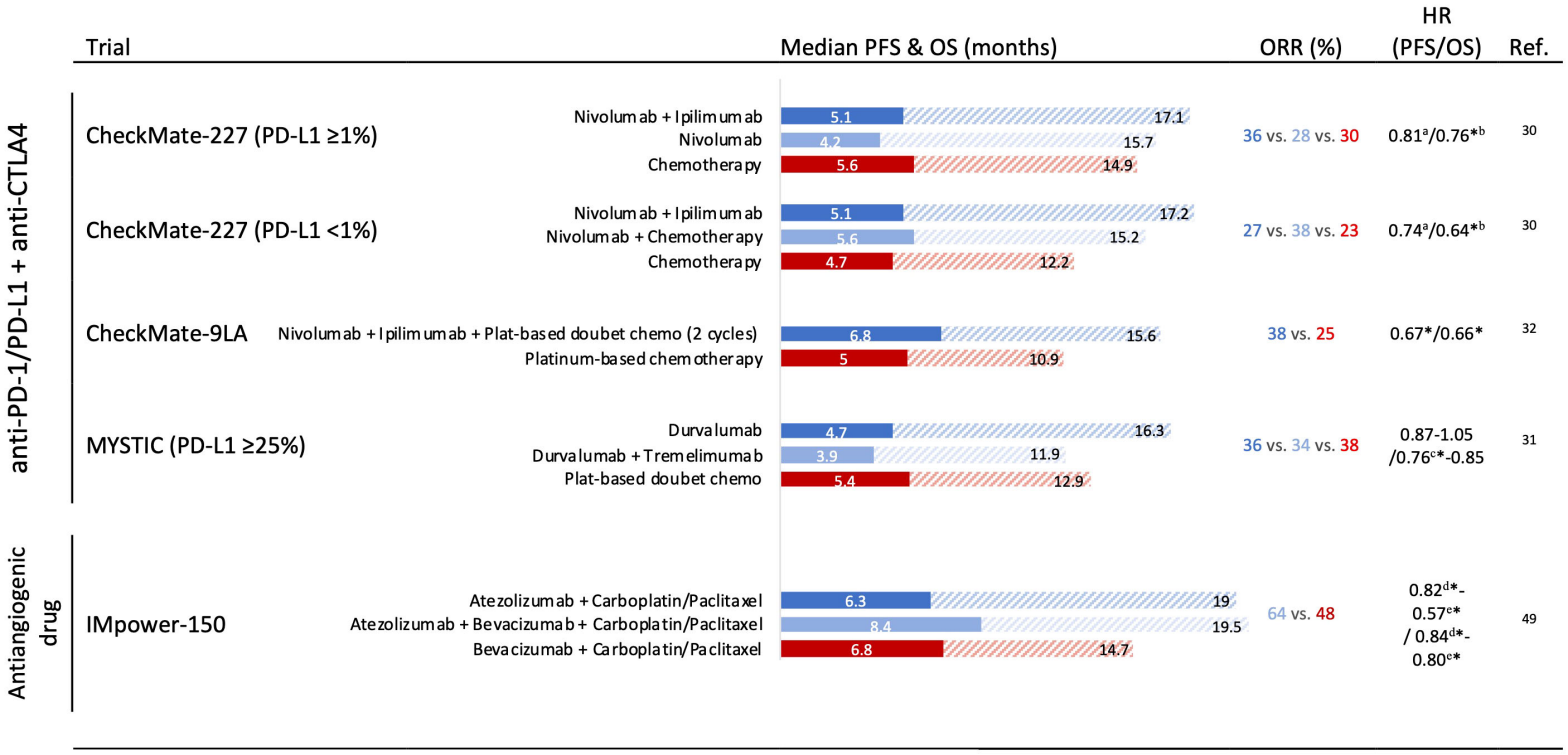
Phase III trials assessing immune checkpoint inhibitor combination and antiangiogenic drug combination strategies in the first-line setting in non-small cell lung cancer with outcomes. HR, hazard ratio; OS, overall survival; PFS, progression-free survival; ORR, overall response rate. ^*^ Significant improvement. ^a^ Significantly improvement of PFS in patients with a high tumor mutational burden (≥10 mutations per megabase). ^b^ Nivolumab + ipilimumab vs. chemotherapy. ^c^ Durvalumab vs. chemotherapy. ^d^ Atezolizumab + carboplatin + paclitaxel *vs*. bevacizumab + carboplatin + paclitaxel. ^e^ Atezolizumab + bevacizumab + carboplatin + paclitaxel vs. bevacizumab + carboplatin + paclitaxel.

The promising results in phase I and II trials using anti-PD-1 plus anti-CTL-4 antibodies led to the evaluation of this dual strategy alone or in combination with chemotherapy in the advanced NSCLC first-line scenario (**Figure 2**). Phase III Checkmate 227 investigated the efficacy of nivolumab alone or in combination with chemotherapy or ipilimumab as first-line therapy in stage IV or recurrent patients with NSCLC. The randomization was performed according to PD-L1-positive or -negative. In both groups, nivolumab plus ipilimumab significantly improved OS compared with chemotherapy alone. Of note, nivolumab plus ipilimumab showed numerically better efficacy compared with nivolumab monotherapy in patients with tumors with PD-L1 expression ≥ 1% and PD-L1 ≥ 50% (30). In this specific exploratory analysis, tumors with PD-L1 ≥ 50% presented 4-year OS rates of 37%, 26%, and 20% with nivolumab plus ipilimumab, nivolumab alone, and chemotherapy alone, respectively.

Notably, in the phase III MYSTIC trial, the combination of durvalumab (anti-PD-L1) plus tremelimumab (anti-CTLA-4) could not improve OS against chemotherapy in PD-L1 ≥ 25% first-line advanced NSCLC (31).

To mitigate the inferior outcomes during the first months when using PD-1 plus CTLA-4 blockade, two trials evaluated the addition of chemotherapy to this regimen. The phase III CheckMate-9LA tested nivolumab plus ipilimumab plus two cycles of chemotherapy demonstrating a significant PFS and OS improvement versus chemotherapy alone in treatment naïve, stage IV, or recurrent NSCLC (**Figure 2**) (32). Similarly, the POSEIDON trial also reported superiority in terms of OS and PFS with first-line durvalumab plus tremelimumab plus chemotherapy versus chemotherapy alone in a recent press release announced (33).

## Combinations with antiangiogenics

Angiogenesis and immunosuppression are both physiological mechanisms involved in nonpathological tissue repair that can be taken advantage of by cancer development and progression (34). Several pro-angiogenic molecules, such as the vascular endothelial growth factor (VEGF), have been linked to a range of immunosuppressive effects at successive steps in the cancer immunity cycle, such as antigen presentation, T-cell priming, T-cell trafficking, and T-cell tumor infiltration (35).

Although blood vessel formation within solid tumors is necessary for cancer survival, tumor abnormal vasculature is characterized by dilated and fragile vessels, which result in leaking, hypoxia, acidosis, and high interstitial pressure. The normalization of this vasculature by specific therapies, such as chemotherapy, irradiation, or especially anti-VEGF antibody, leads to increased T-cell infiltration and therefore enhances tumor immunogenicity (36).

Otherwise, multi-kinase inhibitors such as lenvatinib, cabozantinib, and axitinib, with a preferential antiangiogenic activity, have reported efficacy in combination with anti-PD-1/L1 ICI in some tumor models including renal cell carcinoma and endometrial cancer (37–41). Additionally, bevacizumab plus atezolizumab resulted in positive outcomes in systemic treatment-naive and unresectable hepatocellular carcinoma (42). Although all this evidence supports the combination of ICI and antiangiogenic agents as a successful strategy for some tumor models, previous limited phase I and II trials using this approach reported modest activity in NSCLC (43, 44).

In NSCLC, some trials such as the phase III LEAP-006 evaluate the combination of chemotherapy plus pembrolizumab and lenvatinib in first-line nonsquamous tumors. Preliminary results of the open-label safety run-in (part 1) showed a promising ORR of 69.2% among 13 evaluated patients (45). Additionally, the phase II WJOG @Be study reported encouraging results when testing atezolizumab with bevacizumab for advanced treatment-naive nonsquamous NSCLC with PD-L1 expression ≥50%. In this trial, ORR was 64.1% and median PFS was 15.9 months (46).

Moreover, the phase II Lung-MAP S1800A study testing ramucirumab plus pembrolizumab versus standard of care chemotherapy ± ramucirumab for advanced NSCLC previously treated with immunotherapy demonstrated a significant OS improvement with the combination, whereas no differences were observed in PFS and ORR (22% vs. 28% in combination and standard of care, respectively) (47). Similarly, results from the phase Ib COSMIC-021 were modest when comparing cabozantinib plus atezolizumab (cohort 7) or cabozantinib alone (cohort 20) in patients with advanced NSCLC previously treated with ICIs. In this study, ORR and median PFS were respectively 19% and 4.5 months with the combination, versus 6% and 3.4 months with cabozantinib alone (48).

To date, the most promising was the combination of ICI with antiangiogenic agents and doublet chemotherapy (**Figure 2**). The phase III Impower-150 compared atezolizumab–bevacizumab carboplatin–paclitaxel (ABCP) or atezolizumab–carboplatin–paclitaxel (ACP) versus bevacizumab–carboplatin–paclitaxel (BCP) in nonsquamous metastatic NSCLC. In the intention-to-treat populations, ABCP showed superior PFS and OS compared to BCP (HR 0.57 [0.48–0.67]) and OS (19.5 months vs. 14.7 months; HR 0.80 [0.67–0.95]) (49). However, no differences were observed between ACP and BCP arms. Interestingly, an exploratory analysis showed an OS improvement with ABCP versus BCP in special subgroups with low benefit from ICI monotherapies, such as sensitizing *EGFR* mutations (HR 0.60 [0.31–1.14]), and patients with baseline liver metastases (HR 0.52 [0.33–0.82]) (50).

## Newly emerging co-inhibitory and co-stimulatory checkpoints

The positive clinical impact when using the combination of anti-CTLA-4 and anti-PD-L1 has driven the investigation of other promissory ICI combinations that may increase efficacy. Importantly, resistance to immunotherapy is associated with loss of immunogenic neoantigens, an increase of immunosuppressive cells, and upregulation of alternate immune checkpoint receptors (51). As a consequence, this provides a potential opportunity for novel emerging co-inhibitory and co-stimulatory immune checkpoints.

## TIGIT

T-cell immunoreceptor with immunoglobulin and ITIM domain (TIGIT) is an encouraging new target for cancer immunotherapy. TIGIT is upregulated by immune cells, including activated T cells, natural killer cells, and regulatory T cells. TIGIT binds to two ligands (CD155 and CD112) that are expressed by tumor cells and antigen-presenting cells in the tumor microenvironment (52). Furthermore, TIGIT is coexpressed with PD-1 on exhausted T cells supporting a strong rationale for the dual blockade in restoring T-cell immunity (53). This double inhibition synergizes the proliferation and function of antitumor CD8 T cells, resulting in protective memory T cells and complete tumor rejection (53–55).

Several anti-TIGIT candidate drugs are in development in clinical trials, but tiragolumab is the most advanced. The phase II CITYSCAPE study evaluated tiragolumab plus atezolizumab versus placebo plus atezolizumab as first-line treatment in patients with PD-L1-positive EGFR/ALK wild-type locally advanced or metastatic NSCLC. A higher efficacy was shown with the combination compared with atezolizumab monotherapy (ORR 37% versus 21%, and PFS HR 0.58 [0.39 to 0.88]) (56). A particular benefit was observed in those tumors with PD-L1 ≥ 50% (ORR 66% for combination versus 24% for atezolizumab alone). These findings supported the ongoing phase III SKYSCRAPER-01 with a similar drug arms design, for patients with PD-L1-high locally advanced or metastatic NSCLC. Unfortunately, a recent press release revealed that this trial did not meet the co-primary PFS end point (57).

In addition, a phase I study testing vibostolimab (other anti-TIGIT) showed an ORR of 26% when combined with pembrolizumab in anti-PD-1/PD-L1-naive patients with NSCLC, but minimal efficacy in the anti-PD-1/PD-L1 refractory cohort (ORR 3%) (58).

These results highlight that single anti-TIGIT agents seem not to be an effective strategy, whereas the coadministration with an anti-PD-1/PD-L1 or especially with chemotherapy may be useful and needs to be tested in ongoing clinical trials (NCT04619797, NCT04513925, NCT0495881, NCT04738487, NCT04725188, NCT05226598, NCT05298423, and NCT04165070).

## LAG-3

The transmembrane protein Lymphocyte-activation gene 3 (LAG3, CD223) is an immune inhibitory checkpoint and is expressed on the surface of lymphocytes, such as CD4^+^ T cells, CD8^+^ T cells, natural killer (NK) cells, NK T (NKT) cells, and regulatory T (Treg) cells, which appear when T cells are activated (59–62). The intracellular signaling pathways of LAG3 play a role in the regulation of immune cell function as the coexpression of LAG3 with other inhibitory molecules, including PD-1, TIGIT, TIM-3, 2B4, and CD160, inhibits the tumor immune microenvironment by accelerating T-cell exhaustion and blocking T-cell proliferation (63). The high expression of LAG3 has been associated with unfavorable clinical outcomes in various tumor types including NSCLC (64–66). Furthermore, ICIs can induce resistance through the activation of additional immune checkpoints such as LAG-3 (67).

Since LAG-3 and PD-1 are complementary inhibitory immune checkpoints, dual LAG-3/PD-1 blockade provided a consistent rationale for predicting clinical benefits. In this sense, the combination of the LAG-3-blocking antibody relatlimab and nivolumab has recently revealed a greater benefit in metastatic or unresectable melanoma in the phase II to III RELATIVITY-047 trial (68).

In lung cancer, the combination of eftilagimod alpha, a soluble LAG-3 protein that mediates antigen-presenting cell and CD8 T-cell activation, with pembrolizumab was tested in PD-L1 unselected metastatic NSCLC in the first-line setting (phase II TACTI-002 trial). Among the 36 patients included, response rates by different PD-L1 subgroups were 27% for patients with tumor proportion score (TPS) <1%, 39% for TPS ≥1%, and 54% for ≥50% TPS. Median PFS was 8.2 months while the median OS was not yet reached (69).

Following the favorable evidence in melanoma, current ongoing clinical trials are investigating safety and efficacy of anti-LAG3 drugs in NSCLC (NCT04623775, NCT04205552, NCT04140500, NCT03219268, NCT03365791, NCAGN02385, NCT03849469, NCT02750514, NCT02465060, NCT03780725, NCT03516981, NCT02460224, NCT03250832, NCT01968109, NCT03005782, NCT02966548, and NCT03459222).

## VISTA

V-domain Ig suppressor of T-cell activation (VISTA) is a protein capable of acting as both a ligand and a receptor. VISTA suppresses T-cell proliferation and reduces cytokine production, including IL-10, TNF-α, and IFN-γ (70). Therefore, VISTA blockade can potentially enhance antitumor immune responses. In a phase II pan tumor trial, an oral dual blocker anti-VISTA and PD-L1 agent (CA-170) showed a clinical benefit of 75% and a median PFS of 19.5 weeks among eight previously treated nonsquamous NSCLC patients (71). Of note, several VISTA-targeting inhibitors are being tested in phase I and II trials in patients with metastatic or unresectable solid tumor malignancy including NSCLC (NCT05082610, NCT02671955, and NCT02812875).

## TIM-3

TIM-3 is another inhibitory immune checkpoint molecule similar to CTLA-4 and PD-1. Interaction of TIM-3 with its ligands has been shown to induce T-cell inhibition (72, 73). Interestingly, TIM-3 overexpression has been associated as a negative prognostic marker in NSCLC patients (74). Since the discovery of the negative impact on the immune system by upregulated TIM-3 and PD-L1 coexpression in melanoma, a combination blockade strategy was proposed to restore the T-cell exhaustion (75). The only current clinical data available are a preliminary analysis from the phase I AMBER trial, which included 39 patients with NSCLC who had progressed following initial anti-PD-1 treatment and were tested to receive the anti-TIM-3 antibody cobolimab alone, and in combination with the anti-PD-1 dostarlimab. Of the 20 patients who received the higher dose of cobolimab and were evaluable for response, 3 (15%) had confirmed partial responses and 8 (40%) had stable disease. Notably, all objective responses were among patients with PD-L1 TPS ≥1 (76). Other investigational agents targeting TIM-3 are presently being evaluated in ongoing phase I and II clinical trials enrolling NSCLC patients (NCT03708328, NCT04931654, NCT03652077, NCT03307785, NCT02608268, NCT03099109, NCT03744468, and NCT02817633).

## Co-stimulation

Co-stimulatory immune molecules promote T-cell activation and antitumor immunity. Agonist antibodies against co-stimulatory molecules such as 4-1BB (CD137), OX40 (CD134), and ICOS (CD278) are being investigated in combination with anti-PD-1 agents. However, to date, prohibitive toxicity profiles and modest responses were observed in phase I multi-tumor trials including advanced NSCLC patients (77–82).

## Oncolytic viruses

Oncolytic virus therapy is a novel strategy that promotes immune activation *via* targeted immunogenic cell death. The most developed oncolytic virus T-VEC demonstrated interesting efficacy by injecting intratumorally in patients with melanoma in a phase III study, which led to FDA approval in 2015 (83). However, limited studies evaluated this strategy in lung cancer. Phase Ib KEYNOTE-200 investigated the intravenously delivered oncolytic virus Coxsackievirus A21 (CVA21, CAVATAK) in combination with pembrolizumab in advanced NSCLC and bladder cancer, demonstrating encouraging overall responses of 23% and 33% in 31 ICI-naïve and 21 EGFR/ALK mutation-negative NSCLC patients, respectively (84).

## Targeted therapy

Primarily, the presence of specific oncogene-addicted driver mutations and co-mutations, such as *STK11* and *KEAP1*, has been previously linked to a negative impact on ICI efficacy in NSCLC (85–87).

Preclinical data demonstrated that KRAS-G12C inhibition drives antitumor immunity by enhancing the tumor microenvironment with CD8 T cells, macrophages, and CD103 cross-presenting dendritic cells (88). Consequently, the recent development of direct KRAS-G12C inhibitors has gained interest in the utility of combining KRAS inhibition with immunotherapy, especially for PD-1 refractory *KRAS-STK11* and *KRAS-KEAP1* co-mutated advanced NSCLC. As a consequence, multiple ongoing clinical trials are evaluating KRAS-G12C inhibitors in combination with ICI (NCT03600883, NCT04613596, NCT04449874, NCT04699188, and NCT03785249).

Moreover, based on data from The Cancer Genome Atlas, lung cancer exhibits high levels of homologous recombination deficiency associated with particular mutational signatures. Given these findings, several studies are evaluating PARP inhibitors in combination with chemotherapy and PD-1 blockade in first-line NSCLC (NCT03976323, NCT03976362, and NCT04475939) (89). However, the toxicity profile may still represent a limitation for these combinations since grade ≥3 treatment-emergent adverse events occurred in 88.2% of cases in the phase II JASPER trial evaluating first-line niraparib plus pembrolizumab in patients with advanced NSCLC (90).

## Discussion

ICIs have opened a new era in cancer treatment and particularly for lung cancer. The unprecedented efficacy in NSCLC has begun to resonate with the question of whether the possibility of a cure, at least for a still small subset of patients with advanced disease, is closer. Strong progress has been made in this field, and new challenges for the coming years will be the focus on improving efficacy through a long-term durable response for a larger group of patients. In the course of optimizing the clinical outcomes of ICI in NSCLC, some important steps have substantially impacted patients’ survival, such as the combination of anti-PD-1/L1 with chemotherapy, another ICI, and antiangiogenic agents. Today, multiple strategies are being tested with promising results, from adding different co-inhibitory and co-stimulatory checkpoints, to the combination of ICI with targeted therapy to synergize the anticancer effect.

Altogether this progress was led by a deeper understanding of the defects or alterations in the complex biological relationship processes between the tumor, the microenvironment, and the host, as well as broader insights into the mechanism underlying the resistance of ICI. Regarding the tumor cell-intrinsic features, some areas are of crucial interest beyond the PD-L1 expression as the most studied biomarker in the immunotherapy field. In this context, the study of somatic mutations in the cancer genome that increase tumor mutational and neoantigen burdens has been strongly related to the efficacy of ICI (91). Additionally, multiple efforts are being made to properly characterize the deficiency in neoantigen presentation, aberrations in oncogenes and tumor suppressor genes that regulate immune response (e.g., KRAS, STK11/KEAP1), and the study of genetic alterations in DNA replication and repair genes, epigenetic modulation, and alterations in the interferon-gamma (INF-g) signaling cascade (92). Furthermore, the feature of the tumor microenvironment is now of remarkable interest and is being associated with ICI activity, including the investigation of the phenotype of T-infiltrating lymphocytes, tumor-infiltrating B cells, tertiary lymphoid structures, tumor-associated macrophages, cancer-associated fibroblasts, and endothelial cells. Finally, active investigations are focusing on a comprehensive understanding of the host-related characteristics. Multiple studies have associated the gut microbiome, patient concomitant medications, and autoimmunity with ICI response and/or toxicity (92).

Certainly, as research grows rapidly in this field, the challenge of designing rational and synergistic ICI combination approaches will lead to a lower risk of resistance and prolonged benefits for patient outcomes.

## Author contributions

All authors contributed to the article and approved the submitted version.
